# Nivolumab-Associated Acute Liver Failure: A Report of Two Cases

**DOI:** 10.7759/cureus.106589

**Published:** 2026-04-07

**Authors:** Lekshmi R Shenoi, Saquib Z Banday, Kalpana Acharya

**Affiliations:** 1 Medical Oncology, Sher-i-Kashmir Institute of Medical Sciences, Srinagar, IND; 2 Gastroenterology, Sher-i-Kashmir Institute of Medical Sciences, Srinagar, IND

**Keywords:** acute liver failure, acute liver injury, immune-related adverse events, immune-related hepatic injury, immunotherapy side effects, nivolumab, nivolumab-induced acute liver failure, nivolumab in liver metastasis

## Abstract

Nivolumab is utilized for treating multiple advanced malignancies, with a fraction of patients experiencing immune-related side effects due to immune enhancement. Severe immune-related hepatic injury from nivolumab is very rare and typically responds well to corticosteroids. Here, we present two cases illustrating this phenomenon: one involving extensive-stage small cell carcinoma of the lung with disseminated liver and bone metastasis where nivolumab was administered as a fourth-line treatment and another involving uveal melanoma with liver metastasis where nivolumab was given as first-line palliative systemic therapy. Both cases demonstrated accelerated hepatic deterioration and unresponsiveness to timely steroid administration following the first cycle of nivolumab administration in 3-11 days.

## Introduction

Nivolumab is a monoclonal antibody targeting programmed cell death protein 1 (PD-1) and is widely used in the treatment of various advanced malignancies. By inhibiting PD-1-mediated immune checkpoints, it enhances T-cell activity against tumor cells but can also lead to immune-related adverse events (irAEs) due to loss of self-tolerance.

Approximately 15-25% of patients receiving nivolumab develop immune-related toxicities, including dermatitis, enterocolitis, endocrinopathies, pneumonitis, nephritis, and hepatitis [1-3]. Hepatic involvement is usually characterized by mild to moderate elevations in liver enzymes, with severe immune-related hepatic injury being relatively uncommon [1,3,4]. The onset of hepatotoxicity typically occurs within 1-3 months of therapy and most often demonstrates a hepatocellular pattern, although mixed and cholestatic patterns have also been described [5-7].

Corticosteroids remain the mainstay of treatment for immune-mediated hepatic injury and are generally associated with rapid clinical and biochemical improvement, often allowing for treatment resumption within 1-2 months [8-10]. However, rare cases of severe or steroid-refractory hepatotoxicity have been reported, highlighting the potential for significant morbidity and mortality.

Here, we describe two cases of hyperacute, steroid-refractory hepatocellular injury occurring within days of the first nivolumab dose, both progressing to fulminant hepatic failure. These cases highlight an atypical and aggressive presentation of immune-related hepatotoxicity and underscore the need for early recognition, close monitoring, and better understanding of potential risk factors, particularly in underrepresented populations.

## Case presentation

Case 1

A 41-year-old woman with extensive-stage small cell carcinoma of the lung, previously treated with three lines of chemotherapy (including platinum-based and etoposide-containing regimens), presented with progressive disease in the form of disseminated liver and bone metastases. There was no history of alcohol use, herbal or alternative medicine intake, chronic liver disease, autoimmune disorders, or relevant family history. There was also no history of prior drug-induced liver injury or persistent hepatotoxicity during earlier chemotherapy.

The patient had a good performance status (Eastern Cooperative Oncology Group (ECOG) 1). Baseline evaluation revealed normocytic normochromic anemia, elevated lactate dehydrogenase (LDH), and normal liver and renal function tests, including alkaline phosphatase (ALP). Baseline LDH was elevated at 540 U/L. Inflammatory markers, including procalcitonin (<0.05 ng/mL) and C-reactive protein (CRP) (<5 mg/L), were within normal limits. Autoimmune markers (antinuclear antibody (ANA) and antimitochondrial antibody (AMA)) were negative. The baseline Model for End-Stage Liver Disease (MELD) score was low, consistent with preserved hepatic function before nivolumab initiation.

Nivolumab was administered as fourth-line therapy. On day 5 following the first infusion, the patient presented to the emergency department with jaundice, right upper abdominal discomfort, and anorexia.

On presentation, she was hemodynamically stable, with a blood pressure of 120/80 mmHg, a heart rate of 82 beats per minute, a respiratory rate of 18 breaths per minute, and an oxygen saturation of 98% on room air. She had icterus. Neurological examination revealed that she was conscious but drowsy and oriented to person but not to time and place, with asterixis (flapping tremors), consistent with grade 2 hepatic encephalopathy. There were no cutaneous rashes or mucosal lesions suggestive of immune-related dermatological toxicity. Abdominal examination revealed a soft abdomen with mild right upper quadrant tenderness, without guarding or rigidity. The liver span was 16 cm on clinical examination.

Laboratory evaluation revealed grade 2 anemia and grade 1 thrombocytopenia, with normal total and differential leukocyte counts. Liver function tests showed grade 2 transaminitis (alanine transaminase (ALT) 168 U/L and aspartate aminotransferase (AST) 232 U/L) and grade 3 hyperbilirubinemia (total bilirubin 4.8 mg/dL), as per the Common Terminology Criteria for Adverse Events (CTCAE) v5.0 criteria. Liver function tests and coagulation parameters were monitored serially during hospitalization, with near-daily assessment following clinical deterioration.

Septic screening, including blood cultures, urine analysis and culture, and chest X-ray, was unremarkable. Inflammatory markers remained within normal limits. Viral serology for hepatotropic (hepatitis A, B, C, and E) and non-hepatotropic viruses (cytomegalovirus (CMV), Epstein-Barr virus (EBV), herpes simplex virus (HSV), and dengue) was negative. Autoimmune markers (ANA, AMA) were negative.

Abdominal ultrasound demonstrated hepatomegaly with a liver span of 24 cm and multiple hypo-enhancing lesions in both lobes, with no evidence of vascular thrombosis, ascites, or focal infective source. The discrepancy between clinical (16 cm) and radiological (24 cm) liver span likely reflects limitations of bedside percussion in the presence of marked hepatomegaly due to diffuse metastatic involvement.

**Figure 1:**
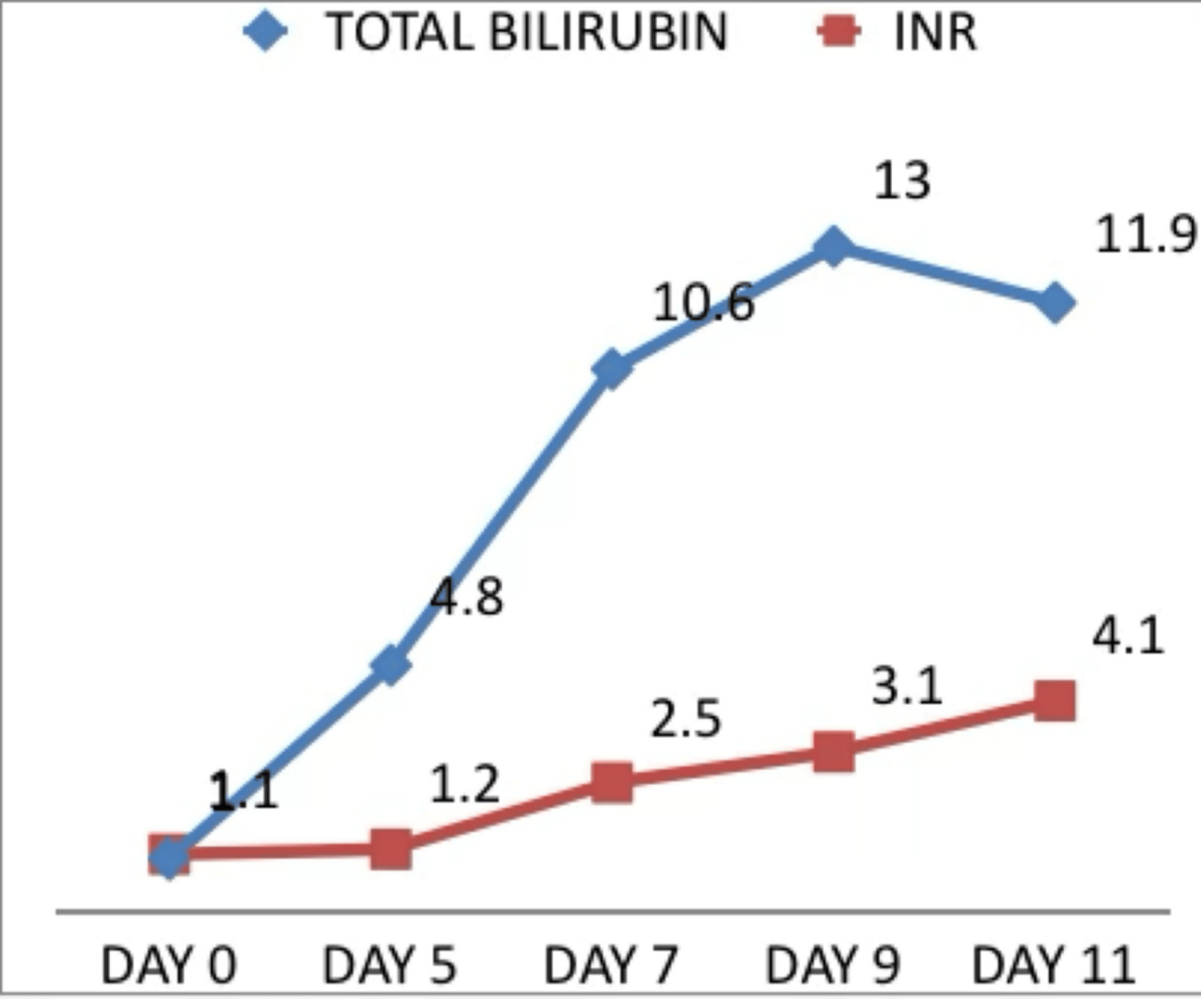
Liver function tests: total bilirubin (mg/dL) and INR INR: international normalized ratio

**Figure 2:**
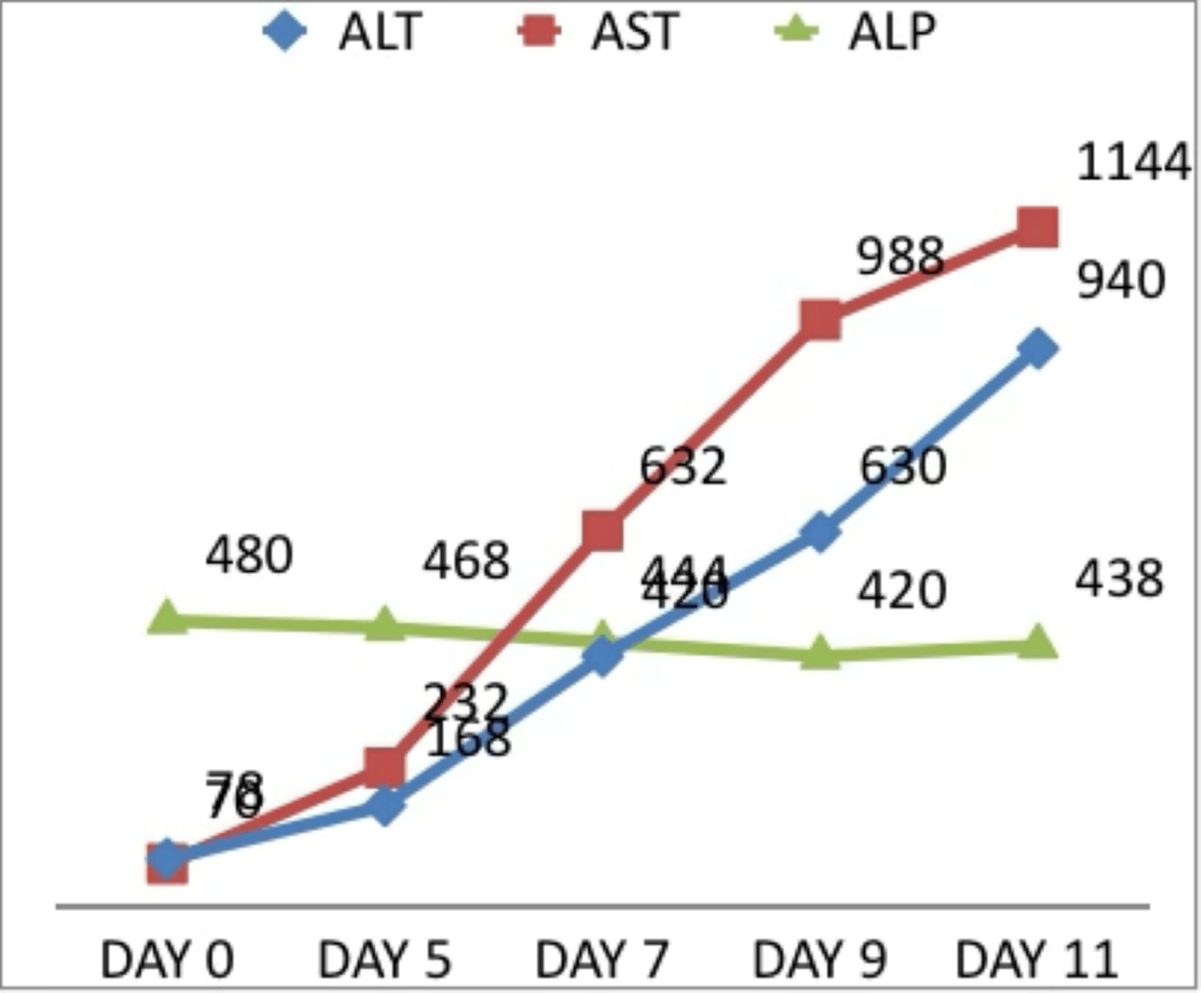
Liver function tests: ALT (U/L), AST (U/L), and ALP (IU/L) ALT: alanine transaminase; AST: aspartate aminotransferase; ALP: alkaline phosphatase

The patient was initiated on intravenous methylprednisolone at a dose of 1 mg/kg/day. Supportive management included intravenous fluids, lactulose, rifaximin, proton pump inhibitors, and anti-edema measures. Despite six days of corticosteroid therapy, liver function tests continued to worsen (Figures 1-2), and the patient progressed to fulminant hepatic failure. The presence of hepatic encephalopathy along with coagulopathy in the absence of pre-existing liver disease fulfilled criteria for acute liver failure.

On day 11 post-nivolumab, the patient exhibited marked deterioration with features of raised intracranial pressure, including bradycardia and unequal pupil size. Fundus examination revealed papilledema. Liver function tests showed grade 4 hyperbilirubinemia (total bilirubin 11.9 mg/dL) and grade 4 transaminitis (ALT 940 U/L and AST 1144 U/L), with relatively lesser elevation of ALP (438 IU/L). Anti-edema measures were initiated, and she was shifted to the intensive care unit; however, she succumbed to fulminant hepatic failure on the same day.

Case 2

A 45-year-old man with uveal melanoma, status post enucleation and radiotherapy, had a disease-free interval of nine months before presenting with progressive disease in the form of liver metastases. There was no history of alcohol consumption, herbal or alternative medication use, prior liver disease, autoimmune conditions, or significant family history.

The patient had a good performance status (ECOG 1). Baseline laboratory evaluation revealed elevated LDH with otherwise normal liver function parameters, including ALP. Baseline LDH was elevated at 646 U/L. Inflammatory markers (procalcitonin and CRP) were within normal limits. Autoimmune markers (ANA and AMA) were negative. The baseline MELD score was low, indicating preserved hepatic function. There was no prior exposure to systemic hepatotoxic chemotherapy.

He received nivolumab as first-line palliative systemic therapy. On day 3 following the first infusion, he presented to the emergency department with altered sensorium.

On presentation, he was hemodynamically stable, with a blood pressure of 118/76 mmHg, a heart rate of 88 beats per minute, a respiratory rate of 20 breaths per minute, and an oxygen saturation of 97% on room air. He had deep jaundice and no meningeal signs. Neurological examination revealed altered sensorium with preserved brainstem reflexes and flexor plantar responses. His Glasgow Coma Scale score was 9 (E2V2M5), consistent with grade 3 hepatic encephalopathy. There were no cutaneous or mucosal findings suggestive of immune-related toxicity. Abdominal examination revealed a soft, non-tender abdomen, with a liver span of 13 cm.

Laboratory evaluation revealed grade 1 anemia and thrombocytopenia. Liver function tests showed grade 4 transaminitis and grade 3 hyperbilirubinemia, with an international normalized ratio (INR) of 5.6 (Table 1). The presence of elevated INR (5.6) along with grade 3 hepatic encephalopathy in a patient without prior liver disease fulfilled the diagnostic criteria for acute liver failure. Electrolytes were within normal limits. Septic screening and inflammatory markers were negative. A non-contrast computed tomography (CT) scan of the head was unremarkable. Viral serology and autoimmune markers (ANA and AMA) were negative.

**Table 1:** Liver function tests ALT: alanine transaminase; AST: aspartate aminotransferase; ALP: alkaline phosphatase; INR: international normalized ratio

Liver function tests	Day 0	Day 3
Total bilirubin (mg/dL)	0.8	12.6
ALT (U/L)	86	1943
AST (U/L)	78	1748
ALP (IU/L)	268	342
Total protein (g/dL)	6.8	6.2
Serum albumin (g/dL)	4.4	3.4
INR	0.8	5.6

The patient was started on high-dose intravenous methylprednisolone (1-2 mg/kg/day), along with supportive management including intravenous fluids, lactulose, rifaximin, and empiric antibiotics. However, he deteriorated rapidly within the same day, progressed to fulminant hepatic failure, and succumbed despite resuscitative measures.

## Discussion

Immune checkpoint inhibitors such as nivolumab act through PD-1 blockade, removing inhibitory signals on T cells and resulting in enhanced immune activation. This can lead to irAEs, including hepatic injury mediated by cytotoxic CD8+ T-cell infiltration and hepatocyte damage.

Approximately 20-30% of patients receiving immunotherapy develop mild to moderate aminotransferase elevations, which are usually self-limited [1,3]. However, only 1-2% progress to severe hepatotoxicity, typically after 2-4 cycles of therapy [1,2]. The pattern of injury is most commonly hepatocellular, although mixed or cholestatic patterns have also been described [5-7].

In both our cases, the presence of coagulopathy and hepatic encephalopathy in the absence of underlying chronic liver disease fulfilled the established criteria for acute liver failure. In contrast to the commonly described timeline, both patients developed hyperacute hepatocellular injury within 3-5 days of the first nivolumab infusion, representing an atypical and aggressive presentation. The biochemical profile was consistent with a hepatocellular pattern, with markedly elevated transaminases and relatively lower ALP levels. Both patients progressed rapidly to fulminant hepatic failure and were clinically refractory to early corticosteroid therapy, suggesting a severe and potentially distinct phenotype of immune-mediated hepatotoxicity.

Causality assessment using the Roussel Uclaf Causality Assessment Method (RUCAM) yielded a score of 5 in both cases, corresponding to a probable likelihood of drug-induced liver injury. This was supported by the strong temporal association with nivolumab, absence of prior hepatotoxic drug exposure, normal baseline liver function, and systematic exclusion of alternative etiologies.

The differential diagnoses considered included acute liver failure due to extensive metastatic infiltration, delayed hepatotoxicity from prior chemotherapy, viral or septic causes, autoimmune hepatitis, and paraneoplastic hepatic dysfunction. However, negative infectious and autoimmune workup, absence of prior hepatotoxicity, and the hyperacute temporal relationship with nivolumab strongly support an immune-mediated etiology.

Although liver biopsy remains the gold standard for diagnosis, histopathological confirmation was not feasible in our cases due to rapid clinical deterioration. Prior studies have demonstrated features of panlobular hepatitis with CD8+ lymphocytic infiltration in immune checkpoint inhibitor-related hepatotoxicity [11,12], supporting a T-cell-mediated mechanism.

It is plausible that patients with pre-existing liver metastases and elevated LDH may have an altered hepatic immune microenvironment, predisposing them to exaggerated immune-mediated injury. Both our patients had liver metastases and elevated LDH, suggesting that these may represent potential baseline risk factors.

Large clinical trials such as ATTRACTION-2 and CheckMate have reported a low incidence of severe hepatotoxicity and rare mortality due to hepatitis [13,14]. In contrast, the hyperacute onset and fulminant course observed in our cases suggest the possibility of a rare but clinically significant phenotype that may be underrecognized in current literature.

Corticosteroids remain the mainstay of treatment for immune-mediated hepatic injury and are generally associated with rapid improvement [1,8-10]. However, both our patients demonstrated clinical refractoriness to early corticosteroid therapy, indicating a severe disease subset. Proposed mechanisms for steroid resistance include persistent immune activation and cytokine dysregulation. In such cases, second-line immunosuppressive agents such as mycophenolate mofetil, tacrolimus, or anti-thymocyte globulin have been described, although evidence remains limited [15,16]. In our setting, escalation of therapy was constrained, particularly during the COVID-19 period.

Both patients demonstrated clinical features of hepatic encephalopathy, further supporting the diagnosis of acute liver failure. Differences between clinical and radiological liver span measurements were also noted, likely reflecting the limitations of bedside percussion in the setting of marked hepatomegaly.

This study has several limitations, including its single-center design, absence of histopathological confirmation, lack of cytokine or immunological profiling, and limited availability of advanced therapeutic options. Nevertheless, the temporal association, biochemical pattern, and exclusion of competing etiologies are consistent with immune checkpoint inhibitor-induced hepatotoxicity as described in existing literature.

Future research should focus on identifying baseline predictors of severe hepatotoxicity, including liver metastases, elevated LDH, and potential genetic or immunological susceptibility. Prospective studies, genomic analyses, and multicenter registries, particularly in underrepresented populations such as the Indian cohort, are needed to better characterize this phenomenon and guide risk stratification and management.

## Conclusions

We describe two cases of hyperacute, steroid-refractory hepatocellular injury following nivolumab therapy, resulting in fulminant hepatic failure. Both cases demonstrated a strong temporal association, hepatocellular pattern of enzyme elevation, and a probable causal relationship based on RUCAM assessment.

These cases highlight a rare but clinically significant presentation of immune-related hepatotoxicity that may occur early in the course of treatment and progress rapidly despite corticosteroid therapy. Early recognition and close monitoring of liver function tests are essential, particularly in patients with baseline liver metastases and elevated LDH, which may represent potential risk factors.

Given the heterogeneity of populations worldwide, genetic and environmental factors may influence both therapeutic response and toxicity profiles. There is a need for more comprehensive data from the Indian population to better characterize the spectrum, severity, and predictors of irAEs.

Further prospective studies, multicenter registries, and pharmacovigilance analyses are required to identify high-risk patients, understand underlying mechanisms, and optimize management strategies for this rare but potentially fatal complication.
